# Top‐down self‐regulation processes as determinants of oral hygiene self‐care behaviour: A systematic scoping review

**DOI:** 10.1002/cre2.548

**Published:** 2022-04-09

**Authors:** Adam A. Rogers, Tiril Willumsen, Hilde Strømme, Jan‐Are K. Johnsen

**Affiliations:** ^1^ Institute of Clinical Dentisty, Faculty of Dentistry University of Oslo Oslo Norway; ^2^ University of Oslo Library University of Oslo Oslo Norway; ^3^ Department of Clinical Dentistry, Faculty of Health Sciences UiT—The Arctic University of Norway Tromsø Norway

**Keywords:** executive function, oral hygiene, review, self‐control, self‐monitoring, self‐regulation

## Abstract

**Objectives:**

Understanding the psychological mechanisms that moderate oral hygiene self‐care behavior is anticipated to benefit efforts to change such behavior. Top‐down self‐regulatory (TSR) processes represent one group of relatively unexplored, yet potentially influential, moderating factors. This systematic scoping review aims to explore whether there is evidence that TSR processes moderate oral hygiene self‐care engagement within the current literature.

**Methods:**

CINAHL, The Cochrane Library, Embase, MEDLINE, PsycINFO, Scopus, and Web of Science databases were searched up to April 2020 for articles that compared measures of TSR processes (such as self‐monitoring, inhibitory control, and task switching) to oral hygiene self‐care behavior, or tested interventions that aimed to change or support TSR processes.

**Results:**

The search returned 6626 articles, with 25 included in the final sample. Weak evidence supported both the role of TSR processes as moderators of interdental cleaning and the value of interventions targeting self‐monitoring of interdental cleaning behavior. Overall, methodological limitations rendered the findings somewhat inconclusive, with an absence of objective assessments of TSR capacity, and little focus on TSR processes as moderators of intervention effects.

**Conclusions:**

The inconclusive, but reasonably promising, findings point to the value of continuing to apply TSR processes within studies of oral hygiene behavior. Exploring why interdental cleaning appears more reliant on TSR processes than toothbrushing, employing objective neuropsychological assessment, and measuring TSR constructs within interventions targeting TSR processes, are encouraged. As a scoping review, the study hopes to generate interest and serve as a starting point for further investigation.

## 1 INTRODUCTION

Top‐down self‐regulatory (TSR) processes are executive functions of the brain that govern the effortful selection and redirection of behavior. While *bottom‐up* processes represent the formation of beliefs, motivations, and associations that increase the salience of a behavioral option (e.g., increasing perceptions of value, adding cue‐associations), *top‐down* processes work to retrieve and compare behavioral options before shifting effort towards a single task or goal (Hofmann et al., 2011, 2012). Without TSR processes, behavior would be heavily reliant on automaticity, leading to engagement solely in actions that are immediately fulfilling or triggered by the immediate environment (Diamond, 2013). This is an important consideration when thinking about preventive health behaviors, as the long‐term focus of preventive actions may mean they require sufficient TSR capacity to facilitate being favored over competing alternatives (Hall & Fong, 2007).

Being synonymous with executive functions, TSR processes typically relate to the cognitive tasks of self‐monitoring, response inhibition, and task switching (Diamond, 2013; Miyake et al., 2000). The role of these processes in health behavior is also highlighted by Temporal Self‐Regulation Theory, which suggests that TSR processes represent the necessary biological capacity required to both ignore unwanted behavioral tendencies and translate positive behavioral intentions into actual engagement (Hall & Fong, 2007). In the oral health field, reduced TSR functionality may explain why a person fails to recall oral hygiene plans, or why they experience difficulties ignoring competing tendencies or redirecting behavior despite knowledge that oral hygiene self‐care will be beneficial.

Although the role of executive functions in health behavior has been advocated (Allan et al., 2016; Gray‐Burrows et al., 2019), few reviews have explored TSR processes in the oral health field. While there is evidence demonstrating the benefits of self‐monitoring interventions (Newton & Asimakopoulou, 2015) and links between conditions such as ADHD (a condition related to difficulties with inhibition and impulsivity) and oral health (Chau et al., 2020), there has been little focus on whether specific TSR processes may contribute significantly to an overall explanation of engagement in preventive oral hygiene self‐care.

Deeper appreciation of the mechanisms that underpin positive health behaviors is expected to benefit attempts to elicit behavioral change (Aklin et al., 2020; Hagger et al., 2020), with understanding the role of TSR processes as moderators of oral hygiene self‐care thus anticipated to help preventive oral health efforts. However, with limited exploration of TSR processes among existing reviews from the oral hygiene field, such a relationship between TSR processes and oral hygiene self‐care is not anticipated to be readily apparent. Establishing whether a relationship exists, therefore, potentially requires broad searching to reveal and assess the applications of TSR constructs within the existing literature. The current study attempts this very goal, taking the form of a systematic scoping review to explore whether existing applications of TSR processes show evidence of moderating engagement in oral hygiene self‐care behavior. The aim is to explore: (i) the role of TSR processes as potential moderators of action and (ii) the methods used to apply TSR processes to the study of oral hygiene behaviors. Analysing these aspects is expected to assist in directing future research and offering conclusions regarding the value of TSR processes within explanatory models of oral hygiene self‐care behavior.

## 2 METHODS

### 2.1 Protocol and registration

A review protocol was designed around the research question: *what evidence is there of a relationship between top‐down self‐regulatory processes and oral hygiene self‐care behaviours*, with the search strategy derived from the PICO statement presented in Table 1. The protocol was registered and published via the Open Science Framework before data collection (https://osf.io/mxkhb/) and the review carried out according to the PRISMA recommendations (Moher et al., 2009).

**Table 1:** Description of the review design in PICO format

PICO item	Definition
Population	Any population.
Intervention/independent variable	Any intervention that explicitly targets top‐down self‐regulatory processes or any quantitative measure of top‐down self‐regulatory capacity.
Comparison	Any quantitative intervention result, or any quantitative comparison.
Outcome	Any quantified measure of oral hygiene self‐care behavior, typically toothbrushing or interdental cleaning.

### 2.2 Eligibility criteria

Studies were selected if TSR constructs were examined as moderators of oral hygiene self‐care behavior, or targeted by an oral hygiene intervention. In either case, studies were required to provide a quantitative statistical comparison and have a full‐text version available in Norwegian, Swedish, Danish, or English language. Psychological and self‐care measures had to be self‐reported or objective measurements pertaining to a single person. This meant that data from caregivers administering oral hygiene care to patients, or parents cleaning the teeth of their children, were excluded. While the review sought to discuss interventions that targeted TSR processes, it did not aim to review specific intervention designs or behavioral change techniques. For example, self‐monitoring and working memory updating represent TSR processes that may be implicitly targeted through keeping a diary or calendar of behavior (Carey et al., 2019). However, the review did not aim to collect information pertaining to all uses of dairy interventions, only those that mentioned targeting TSR processes—that is, self‐monitoring or working memory.

### 2.3 Search

The following databases were searched on April 2nd, 2020: CINAHL (EBSCO), The Cochrane Library (Wiley), Embase (Ovid), MEDLINE (Ovid), PsycINFO (Ovid), Scopus, Web of Science. No limit was set on date, language, or type of publication during search phase. The electronic search strategy was created via preliminary searching, extraction of keywords from existing reviews, and consultation with two psychologists. A variety of synonyms were used to capture TSR mechanisms, including terms related to self‐referent cognitions, monitoring and inhibition, and general executive functions. Oral hygiene self‐care terms incorporated general phrases related to oral health and behavioral frequency. Relevant subject headings for each category were also included. A full copy of the electronic search strategy is included in Appendix A.

All search results were exported to EndNote and duplicates were removed using the methods prescribed by Bramer (2016). Titles and abstracts were screened by two reviewers (AAR & HS) using Rayyan software (Ouzzani et al., 2016) and a screening protocol of inclusion/exclusion criteria. Following preliminary screening, full‐text analysis was conducted by three members of the research team (AAR, TW, J‐AKJ) to determine suitability for inclusion in the final sample with consensus reached for all included studies.

### 2.4 Data collection

Data were extracted through collaboration between three reviewers (AAR, TW, J‐AKJ). To address the research question regarding the relationship between TSR processes and oral hygiene self‐care, data items included the instruments used to assess oral hygiene behavior and TSR constructs, the methods used within intervention processes, and the observed statistical relationships or effects. Where data were unavailable, corresponding authors were contacted.

## 3 RESULTS

**Figure 1:**
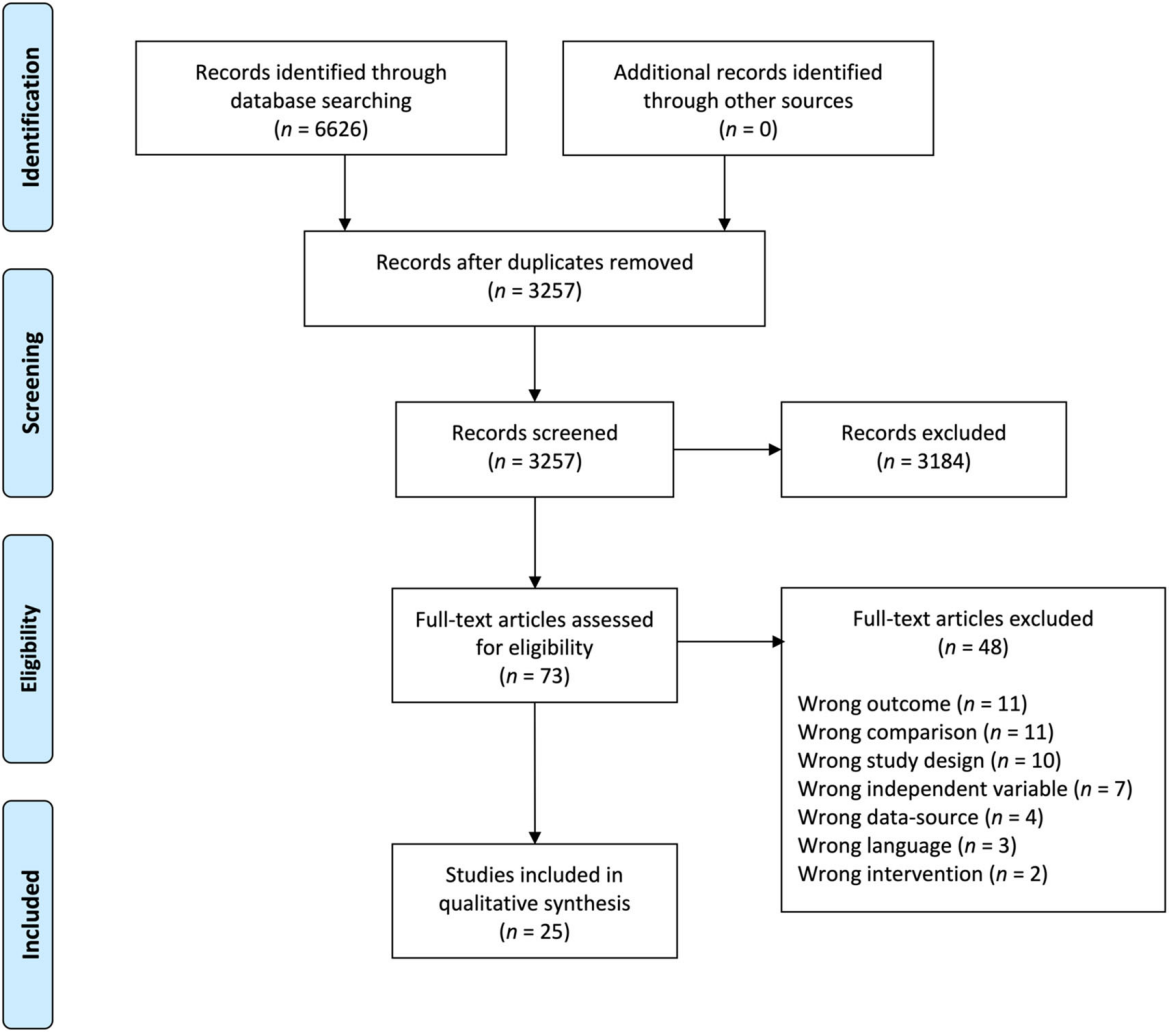
Flow chart of the review process

The initial search returned 6626 results. After duplicate removal and screening of the remaining 3257 unique articles, 73 advanced to full‐text review and the final sample included 25 studies and 25 unique populations. A flow‐chart of the review process is presented in Figure 1 and demographic information presented in Table 2. Details of the studies excluded during full‐text review are provided in Appendix B. The samples included participants from 13 different countries, with a median sample size of 151 participants, and with 60% of samples taken from school or university students. The included studies showed a distinct preference to focus on either plaque removal via toothbrushing or plaque removal via the use of interdental cleaning aids. Extracted data were therefore grouped based on study design (cross‐sectional or intervention) and target behavior (toothbrushing or interdental cleaning), and is presented in Tables 3, 4, 5, 6.

**Table 2:** Demographic information for the included studies

Author (year)	Country	*N*	Age group	Participants	Recruiting	
McGlynn et al. (1985)	USA	52	NR	University students	Cohort of second year dental students	1
McGlynn et al. (1987)	USA	59	16.3 (NR)	Dental patients	Patients invited to volunteer	2
O'Neill et al. (1987)	USA	25	NR	University students	Recruiting not specified	3
Stewart et al. (1991)	USA	100	Range: 21–65	Hospital patients	Patients invited to volunteer	4
McCaul et al. (1992) (Exp 2)	USA	77	30.2 (NR)	University students	University organization members invited by mail	5
McCaul et al. (1992) (Exp 1)	USA	38	NR	University students	Cohort of first year psychology students	6
Little et al. (1997)	USA	107	56.9 (NR)	Dental patients	Patients invited to volunteer	7
Sniehotta and Schüz (2006)	Germany	470	NR	Dental patients	Patients invited to volunteer	8
Schuz (2007)	Germany	151	25.15 (6.99)	University students	Cohorts of psychology and education students	9
A. L. Dumitrescu et al. (2009)	Romania	178	19.11 (1.43)	University students	Cohort of first year medical students	10
A. L. Dumitrescu et al. (2010)	Romania	213	19.26 (1.37)	University students	Cohort of first year dental students	11
A. L. Dumitrescu et al. (2011)	Romania	212	19.26 (1.38)	University students	Cohort of first year dental students	12
A. L. Dumitrescu et al. (2011)	Romania	198	19.75 (1.35)	University students	Cohort of first year medical students	13
A. L. Dumitrescu et al. (2012)	Romania	205	29.84 (9.78)	Dental patients	Existing patients from a dental clinic	14
Suresh et al. (2012)	Kuwait	53	33.55 (7.20)^a^	Dental patients	Patients invited to volunteer	15
Ein‐Gar et al. (2012)	Israel	111	26 (NR)	University students	Recruiting not specified	16
Naorungroj et al. (2013)	USA	8782	56.8 (5.7)	Community sample	Cohort of adults from a cardiovascular disease epidemiology study	17
Lhakhang et al. (2015)	India	205	20.7 (1.59)	University students	Recruited via advertisment boards	18
Zhou et al. (2015)	China	215	21.35 (1.39)	University students	Recruiting not specified	19
Schwarzer et al. (2015)	Poland	287	21.36 (1.55)	University students	Recriuted via advertisment boards	20
Pakpour et al. (2016)	Iran	1109	15.35 (1.32)^a^	School students	Class cohorts within 48 indivdiual schools invited to participate	21
Hamilton et al. (2018)	Australia	241	22.23 (6.40)	University students	Recruiting not specified	22
Asimakopoulou et al. (2019)	England	97	60.61 (11.24)	Dental patients	Patients invited to volunteer	23
Araújo et al. (2019, 2020)	Portugal	201	38.6 (12.49)	Community sample	Advertisments in local newspapers advertising boards and dental clinics	24
Scheerman et al. (2020)	Netherlands	132	13.35 (0.99)^a^	Dental patients	Patients invited to volunteer	25

^a^ Average of split sample.

**Table 3:** Relationships between TSR processes and toothbrushing frequency in cross‐sectional studies

Author	*N*	Toothbrushing measure	Independent variable	Results
A. L. Dumitrescu et al. (2009)	178	Daily frequency, ordinal scale	Self‐Control Scale—Brief Source: Tangney et al. (2004)	○○
A. L. Dumitrescu et al. (2010)	213	Daily frequency, ordinal scale	Behavioural Inhibition System Source: Carver and White (1994)	○○
A. Dumitrescu et al. (2011)	198	Daily frequency, ordinal scale	Self‐Regulation Scale Source: Schwarzer et al. (1999)	○○
A. L. Dumitrescu et al. (2011)	212	Daily frequency, ordinal scale	Focus of Attention Questionnaire Source: Woody (1996)	○○
A. L. Dumitrescu et al. (2012)	205	Daily frequency, ordinal scale	Self‐Control Scale—Brief Source: Tangney et al. (2004)	○○
Naorungroj et al. (2013)	8782	Daily frequency, ordinal scale	Executive functions: Delayed Word Recall Test. Source: Knopman and Ryberg (1989). Word Fluency Test Source: Benton et al. (1981). Digit Symbol Substitution Test Source: Wechsler (1981)	●○
Pakpour et al. (2016)	1109	Monthly frequency, interval scale	Self‐monitoring Source: None provided	●●
Araujo et al. (2020)	201	Daily frequency, ordinal scale	Action control Source: Godinho et al. (2014)	○○

Abbreviations: ○, No relationship detected; ●, Relationship at single timepoint or in group comparison; ●●, Significant linear relationship; TSR, top‐down self‐regulatory.

**Table 4:** Relationships between TSR processes and interdental cleaning frequency in cross‐sectional studies

Author	*N*	Interdental cleaning measure	Independent variable	Results
O'Neill et al. (1987)	25	Flossing, weekly frequency, ordinal scale	Covert self‐monitoring Source: None provided	●○
Schuz et al. (2007)	151	Flossing, monthly frequency, interval scale	Action control Source: Sniehotta et al. (2005)	●●
A. L. Dumitrescu et al. (2009)	178	Flossing, monthly frequency, ordinal scale	Self‐Control Scale—Brief Source: Tangney et al. (2004)	○○
A. L. Dumitrescu et al. (2010)	213	Not speficied	Behavioural Inhibition System Source: Carver and White (1994)	●○
A. L. Dumitrescu et al. (2011)	198	Not speficied	Self‐Regulation Scale Source: Schwarzer et al. (1999)	○○
Suresh et al. (2012)	53	Flossing, weekly frequency, ordinal scale	Action control. Source: Schuz et al. (2007)	●●
Ein‐Gar et al. (2012)	111	Flossette use, daily frequency, interval scale^a^	Dispositional Self‐Control Source: Ein‐Gar et al. (2008), Ein‐Gar and Steinhart (2011)	○○
A. L. Dumitrescu et al. (2012)	205	Flossing, monthly frequency, ordinal scale	Self‐Control Scale—Brief Source: Tangney et al. (2004)	●○
Naorungroj et al. (2013)	8782	Flossing, weekly frequency, ordinal scale	Executive functions: Delayed Word Recall Test Source: Knopman and Ryberg (1989). Word Fluency Test Source: Benton et al. (1981). Digit Symbol Substitution Test Source: Wechsler (1981)	●○
Schwarzer et al. (2015)	287	Flossing, daily frequency, interval scale	Self‐monitoring Source: None provided	●●
Hamilton et al. (2018)	241	Flossing, weekly frequency, hybrid scale^b^	Action Control Source: None provided	●●
Araújo et al. (2020)	201	Flossing, daily frequency, ordinal scale	Action control Source: Godinho et al. (2014)	●○

Abbreviations: ○, no relationship detected; ●, relationship at single timepoint or in group comparison; ●●, significant linear relationship; TSR, top‐down self‐regulatory.

^a^ Scale objectively measured.

^b^ Scale was a factor derivative of responses to an ordinal and interval scale.

**Table 5:** Effect of targeting TSR processes on toothbrushing frequency within intervention studies

Author	*N* (*T*)	Intervention delivery format	Brushing measure	Intervention content	Intervention type	Results
McGlynn et al. (1985)	52 (NR)	Face‐to‐face, single goal‐setting session followed up after 2 weeks	Weekly frequency, ordinal scale	Daily self‐monitoring of brushing behavior for 2 weeks after receiving goal‐setting instructions focused on other behaviors	Self‐monitoring	○○○
McGlynn et al. (1987)	59 (29)	Face to face, single session, followed up after 2, 5, and 8 weeks	Weekly frequency, interval scale	Self‐management intervention including a booklet that discussed goal‐setting, self‐evaluation, and self‐monitoring of daily behavior for 8 weeks	Self‐monitoring plus goal‐setting materials	○○○
Stewart et al. (1991)	100 (25)	Face‐to‐face, 20‐min educational session, and two 25‐min CBI sessions at one‐week intervals. Followed up 2 weeks after last session	Weekly frequency, interval scale	Cognitive‐behavioral intervention targeting education, anticipated consequences, planning for barriers, and self‐monitoring of daily behavior for 1 week between CBI sessions	Self‐monitoring plus motivation and goal‐setting	●●○
Little et al. (1997)	107 (54)	Face‐to‐face, five 90‐min sessions spaced 1 week apart, followed up 4 months after the first session	Weekly frequency, interval scale	Five 90‐min oral hygiene classes. Classes targeted feedback on outcomes of behavior, social comparison, behavioral practice, goal‐setting, and self‐monitoring of daily behavior for 4 months	Self‐monitoring plus motivation and goal‐setting	●●○
Pakpour et al. (2016)	1109 (367)	Written intervention, single session, followed up at 1 and 6 months	Monthly frequency, interval scale	Leaflet containing educational information and instructions to self‐monitor daily behavior (timeframe not specified)	Self‐monitoring & education	○○○
Asimakopoulou et al. (2019)	97 (33)	Face‐to‐face, single session, followed up at 4 and 12 weeks	Weekly frequency, interval scale	Goal‐setting, planning, and self‐monitoring intervention. Included risk assessment, goal‐setting session, and self‐monitoring of daily behavior for 12 weeks	Self‐monitoring plus motivation and goal‐setting	○○○
Scheerman et al. (2020)	132 (67)	Digital intervention, active for 12 weeks	Daily frequency, interval scale	Smartphone application targeting self‐monitoring of outcomes, education about consequences, goal‐setting, implementation intentions, behavioral reminders, support messages, coping planning, and self‐monitoring of daily behavior for 12 weeks	Self‐monitoring plus motivation and goal‐setting	○○○

Abbreviations: ○, No effect; ●, Effect registered but no control comparison; ●●, More effective than control but not better than alternative treatment; ●●●, More effective than comparative treatment; CBI, cognitive‐behavioral intervention.

**Table 6:** Effect of targeting TSR processes on interdental cleaning frequency within intervention studies

Author	*N* (*T*)	Intervention delivery format	Brushing measure	Intervention content	Intervention type	Results
McGlynn et al. (1985)	52 (NR)	Face‐to‐face, single goal‐setting session targeted at alterior actions followed up after 2 weeks of self‐monitoring	Weekly frequency, ordinal scale	Daily self‐monitoring of brushing behavior for 2 weeks after receiving goal‐setting instructions focused on other behaviors	Self‐monitoring	○○○
Stewart et al. (1991)	100 (25)	Face‐to‐face, 20‐minute educational session and two 25‐minute CBI sessions at one‐week intervals with one week of self‐monitoring in between. Followed up 2 weeks after last session	Flossing, weekly frequency, interval scale	Cognitive‐behavioral intervention targeting education, anticipated consequences, planning for barriers, and self‐monitoring of daily behavior for one week between CBI sessions	Self‐monitoring plus motivation and goal‐setting	●●○
McCaul et al. (1992)	45 (15)	Face‐to‐face, sessions at beginning and 4 weeks to provide materials to complete the 10‐week intervention. Followed up at conclusion of intervention	Flossing, weekly frequency, ordinal scale	Education and motivation intervention followed 4 weeks later by self‐monitoring intervention. Calendar used to self‐monitor daily behavior for 10 weeks	Self‐monitoring plus motivation	○○○
McCaul et al. (1992)	45 (15)	Face‐to‐face. One session at the beginning of self‐montioring, and another at 4 weeks to provide motivation and feedback. Followed up at the conclusion of the 10‐week intervention	Flossing, weekly frequency, ordinal scale	Education and motivation intervention followed 4 weeks later by self‐monitoring intervention. Ten‐week daily self‐monitoring intervention contained a mid‐way session for skills‐training, feedback and action planning, as well as fortnightly prompts via telephone	Self‐monitoring plus motivation and goal‐setting	○○○
McCaul et al. (1992)	77 (24)	Face‐to‐face, sessions at beginning and 4 weeks to provide materials to complete the 10‐week intervention. Followed up at conclusion of intervention	Flossing, weekly frequency, ordinal scale	Intervention regarding flexible goal‐setting given 2 weeks before self‐monitoring intervention. Self‐monitoring intervention focussed on monitoring of daily behavior for 8 weeks	Self‐monitoring plus goal‐setting	○○○
McCaul et al. (1992)	77 (30)	Face‐to‐face, sessions at beginning and 4 weeks to provide materials to complete the 10‐week intervention. Followed up at conclusion of intervention	Flossing, weekly frequency, ordinal scale	Intervention regarding difficult goal‐setting given 2‐weeks before self‐monitoring intervention. Self‐monitoring intervention focussed on monitoring of daily behavior for 8 weeks	Self‐monitoring plus goal‐setting	●●●
Little et al. (1997)	107 (54)	Face‐to‐face, five 90‐min sessions spaced 1 week apart, followed up at conclusion of 4‐month self‐monitoring intervention	Weekly frequency, interval scale	Five 90‐minute oral hygiene classes. Classes targeted feedback on outcomes of behavior, social comparison, behavioral practice, goal‐setting, and self‐monitoring of daily behavior for 4 months	Self‐monitoring plus motivation and goal‐setting	●●○
Sniehotta and Schüz (2006)	470 (147)	Written intervention, single exposure with 4 weeks of self‐monitoring, followed up at conclusion of intervention	Flossing, weekly frequency, interval scale	Self‐regulation intervention focussing on education, goal‐setting, and self‐monitoring of behavior for 4 weeks	Self‐monitoring plus goal‐setting	○○○
Schuz et al. (2007)	151(151)	Written intervention, single exposure with 2 weeks of self‐monitoring. Followed up 4 weeks after the conclusion of the self‐monitoring intervention	Flossing, monthly frequency, interval scale	Self‐monitoring intervention. Calendar used to self‐monitor daily behavior for 2 weeks	Self‐monitoring	●○○
Suresh et al. (2012)	53 (53)	Written intervention, single exposure, followed up at conclusion of 4‐week self‐monitoring intervention	Flossing, weekly frequency, ordinal scale	Self‐monitoring intervention. Calendar used to self‐monitor daily behavior for 2 weeks	Self‐monitoring	●○○
Lhakhang et al. (2015)	205 (94)	Written intervention, single exposure delivered 17 days after a written motivational intervention and followed up at conclusion of a 17‐day self‐monitoring intervention	Flossing, daily frequency, interval measure	Initial motivational intervention focussed on education, outcomes, costs and benefits, and intention‐formation. After 17 days, the self‐regulation intervention was delivered and focussed on goal setting, coping strategies, goal review, and self‐monitoring of daily behavior for the following 17 days	Self‐monitoring plus motivation and goal‐setting	●●●
Lhakhang et al. (2015)	205 (111)	Written intervention, single exposure, followed up at the conclusion of a 17‐day self‐monitoring intervention	Flossing, daily frequency, interval measure	Self‐regulation intervention focussed on goal‐setting, coping strategies, goal‐review, and self‐monitoring of daily behavior for 17 day	Self‐monitoring plus goal‐setting	●●●
Schwarzer et al. (2015)	144 (48)	Written intervention, single exposure, followed up at conclusion of 3‐week self‐monitoring intervention	Flossing, daily frequency, interval measure	Self‐regulation intervention targeting self‐belief, action planning, and self‐monitoring of daily behavior for 3 weeks	Self‐monitoring plus goal‐setting	○○○
Zhou et al.(2015)	215 (127)	Written intervention, single exposure, followed up after 1 month	Flossing, daily frequency, interval measure	Self‐regulation intervention focussed on education, goal‐setting, and self‐monitoring daily behavior for 1 month	Self‐monitoring plus motivation and goal‐setting	●●○
Asimakopoulou et al. (2019)	97 (33)	Face‐to‐face, single session, followed up at 4 weeks and at conclusion of 12‐week self‐monitoring intervention	Morning interdental cleaning, weekly frequency, interval scale	Goal‐setting, planning, and self‐monitoring intervention. Included risk assessment, goal‐setting session, and self‐monitoring of daily behavior for 12 weeks	Self‐monitoring plus motivation and goal‐setting	●●○
Asimakopoulou et al. (2019)	97 (33)	Face‐to‐face, single session, followed up at 4 weeks and at conclusion of 12‐week self‐monitoring intervention	Evening interdental cleaning, weekly frequency, interval scale	Goal‐setting, planning, and self‐monitoring intervention. Included risk assessment, goal‐setting session, and self‐monitoring of daily behavior for 12 weeks	Self‐monitoring plus motivation and goal‐setting	●●●
Scheerman et al. (2020)	132 (67)	Digital intervention, active for 12 weeks	Interproximal brushing, monthly frequency, ordinal scale	Smartphone application targeting self‐monitoring of outcomes, education about consequences, goal‐setting, implementation intentions, behavioral reminders, support messages, coping planning, and self‐monitoring of daily behavior for 12 weeks	Self‐monitoring plus motivation and goal‐setting	○○○

Abbreviations: ○, no effect; ●, effect registered but no control comparison; ●●, more effective than control but not better than alternative treatment; ●●●, more effective than comparative treatment; CBI, cognitive‐behavioral intervention.

### 3.1 Findings from cross‐sectional studies

Table 3 presents data from the studies (*n* = 8) that examined the relationship between TSR processes and toothbrushing frequency. Overall, little evidence suggested a significant relationship. However, it should be noted that only one study attempted neuropsychological assessment of executive functions instead of self‐report methods (Naorungroj et al., 2013), and only one explored variations in toothbrushing beyond daily frequency (Pakpour et al., 2016), with both studies observing stronger relationships.

Table 4 presents data from the studies (*n* = 12) that tested the presence of a relationship between TSR processes and flossing frequency. In contrast to the toothbrushing results, the evidence pertaining to interdental cleaning was more indicative of a potential relationship. The presence of linear relationships, however, was mixed. Only four linear relationships were reported, with three of these between flossing and action control (Hamilton et al., 2018; Schuz et al., 2007; Suresh et al., 2012). Among the flossing studies, there was a reduced tendency to use daily‐frequency as the timeframe for behavior with studies favoring weekly or monthly recall periods.

### 3.2 Findings from intervention studies

Looking at the intervention studies, the data from those that tested TSR interventions on toothbrushing frequency is presented in Table 5. Again, there was little evidence that TSR‐focussed interventions influenced toothbrushing frequency. Regarding the interventions themselves, all targeted the processes of self‐monitoring/memory‐updating via the use of behavioral diaries, with the logging of behavior anticipated to increase the effort dedicated to monitoring whether behavior was completed or not. Periods of diary‐use ranged from 1 week to 4 months, with all studies except one combining the self‐monitoring facet with additional behavioral change techniques. Within the single study that isolated the effect of the diary, no significant effect was detected (McGlynn et al., 1985).

Data from the intervention studies that examined interdental cleaning are presented in Table 6. Again, all interventions targeted self‐monitoring through the use of diary interventions, with the relationship between self‐monitoring interventions and interdental cleaning the most widely explored within the unique samples (*k* = 16). It should be noted that none of the included studies utilized interventions related to alternative TSR processes, such as training metacognitive self‐awareness, response inhibition, or attention control. Within the studies of interdental cleaning, three isolated the effect of a self‐monitoring program, with two reporting positive results (Schuz et al., 2007; Suresh et al., 2012). In 10 out of 17 applications the results suggested a relationship between the use of self‐monitoring interventions and increased interdental cleaning behavior.

Overall, the results demonstrated that top‐down processes were more related to interdental cleaning behavior than toothbrushing behavior and that self‐monitoring interventions may have a positive influence on interdental cleaning frequency. Among the methods, there was considerable heterogeneity in the measures used to quantify oral hygiene self‐care and TSR processes, as well as heterogeneity in the design of TSR‐focussed interventions. Although the scoping review did not plan to offer rigid comparisons, the apparent general heterogeneity points to some interesting considerations for future study that are discussed below.

## 4 DISCUSSION

This systematic scoping review aimed to explore whether TSR processes play a role in the moderation of oral hygiene self‐care behaviors. Based on the current findings, interdental cleaning appears to have a stronger relationship with TSR processes, although due to varied methodological limitations and incomparable construct definitions these findings are somewhat inconclusive. As a scoping review, inconclusive results were neither unexpected nor fruitless. Rather, the results point to interesting gaps in the literature and potential pathways for the continued application of TSR processes within future research.

### 4.1 Between‐behavior differences in association with TSR processes

A key finding from the current review was the differences in association strength based on the behavior in focus. TSR processes, in general, appeared to be more associated with interdental cleaning than with toothbrushing. As executive functions are associated with a range of health behaviors (Gray‐Burrows et al., 2019; Reimann et al., 2020), this difference was not anticipated. One explanation is that interdental cleaning may be perceived as more challenging to perform than toothbrushing, with reliance on executive resources increasing with task difficulty (Tun & Lachman, 2008). However, alternative explanations might also relate to the automaticity of toothbrushing behavior or the time‐perspective of outcomes linked to interdental cleaning. Automaticity increases with behavioral familiarity, allowing for increased unconscious processing and reducing demand on top‐down self‐regulatory control (Reisberg, 2013). As toothbrushing is usually emphasized more than interdental cleaning from an early age, it may be predisposed to greater implicit familiarity and automaticity, meaning an increased possibility of engagement in the absence of conscious self‐regulatory effort. Hall and Fong (Hall & Fong, 2007) refer to this quality as *behavioural prepotency*, suggesting that implicit tendencies towards a behavior, based on past familiarity, increase the likelihood of action and present a moderating factor that acts independently of explicit intentions and executive function capacity. Regarding time‐perspective, the temporal proximity of reward outcomes is suggested to influence how competing actions are weighed against one another (Hall & Fong, 2007). As toothbrushing is associated with a greater total plaque removal efficacy than interdental cleaning (Terezhalmy et al., 2005; Terézhalmy et al., 2008), it is likely that the temporally *proximal* reward, that is, the immediate benefit, is perceived to be greater, despite the temporally *distal* rewards (e.g., the effect on periodontitis risk), being more similar. Lower immediate benefits are suggested to negatively impact the likelihood that interdental cleaning is favored over competing for alternative behaviors, increasing the reliance on top‐down self‐regulatory capacity to facilitate engagement (Hall & Fong, 2007). Although task difficulty, automaticity, and time‐perspective were not explored within the current studies, they may represent key factors that explain the observed differences between unique oral hygiene self‐care behaviors and their relationships with TSR processes.

### 4.2 Interpretation of intervention effects

The results also point to the mixed effects of self‐monitoring interventions, with slightly more frequent positive results observed among interdental cleaning studies. Explaining mixed results may have to do with TSR processes themselves. Within studies of dieting, executive planning capacity has been shown to moderate the effect of planning interventions (Allan et al., 2013). This means that a planning intervention may not register an effect if the individual is already adept at cognitive planning. Within the current studies, this moderating effect was unable to be explored with only two experimental self‐monitoring interventions attempting to measure self‐monitoring capacity (Pakpour et al., 2016; Schwarzer et al., 2015). Another explanation for the mixed results may have been the design of the intervention materials. In addition to considerable heterogeneity in the time‐span of the interventions (range: 1 week to 4 months), there was little discussion about how the diaries were actually used. It is anticipated that while entering into an agreement to record one's behavior may increase the attention and self‐monitoring of behavior, placing a diary on the bathroom counter may have a different effect as a prompting device. This presents potential for diaries to work as both top‐down (encouraging self‐monitoring and metacognitions) and bottom‐up (providing accessible environmental triggers) interventions. Further research is required to separate and understand the true effects of diary interventions as a behavioral change technique.

### 4.3 Strengths and challenges

The current study did have considerable strengths as a novel scoping review concerning a relatively unexplored pathway within the field of preventive oral health. Namely, the review aimed to be broad and impartial, to incorporate learning from the behavioral sciences, and to employ a systematic and inclusive search strategy that avoided testing any particular theory or approach. However, it should be noted that defining where top‐down and bottom‐up processes differentiate is a topic of continued debate (Evans & Stanovich, 2013). Thus, the selection criteria should be interpreted as an attempt to include higher‐order functions occurring within close temporal proximity to the behavior itself, and a reflection of executive functions associated with task control in the wider literature (Miyake et al., 2000).

This inclusivity, though, resulted in rather inconclusive findings; there were few linear relationships between TSR processes and oral hygiene behavior, and an inability to generalize them based on the apparent heterogeneity. An interesting observation was that three of the five linear relationships involved the construct of *action control* (Hamilton et al., 2018; Schuz et al., 2007; Suresh et al., 2012), a construct that attempts to capture general capacity for effortful top‐down self‐regulation. Stronger relationships with broader construct definitions, but not with more specific ones, provides some evidence that while a relationship may indeed exist between TSR processes and oral hygiene behavior, there may be inherent difficulties in isolating and exploring the underlying sub‐processes themselves.

One reason for such difficulties can be explained by the methods used to quantify TSR processes. Within the current studies, for example, only one attempted neuropsychological assessment (Naorungroj et al., 2013). Neuropsychological assessment involves testing performance on a behavioral task, with measures shown to differentiate considerably from self‐report (Saunders et al., 2018) and with neuropsychological assessment favored within theoretical behavior models that include TSR processes (Hall & Fong, 2013). It is plausible that using objective cognitive assessment may tell a different narrative to the one observed in the present study. Similarly, the study found that measures used to quantify oral hygiene self‐care could also benefit from refinement. With weaker relationships generally observed when behavior was measured on a times‐per‐day basis, it is recommended that recall periods be extended to a weekly timeframe. Not only do daily measures also have a tendency to mirror internalized habits (Hagger et al., 2015), rather than actual behavior, but weekly recall periods offer a reasonably valid approximation that is likely to better estimate the real variability in the target action (Stull et al., 2009).

### 4.4 Future directions

Overall, the current review may be used as a guide for several novel research pathways. First, future research may consider neuropsychological testing as a means of continuing research into TSR process within the oral health field. With objective measures potentially likely to tell a different narrative, applying neuropsychological assessment and a theoretical framework that includes TSR processes, such as Temporal Self‐Regulation Theory (Hall & Fong, 2007), represents an ideal starting point. Second, experimental studies may consider similar tactics. As evidenced in the health field, executive functions may moderate the influence of behavior‐change interventions (Allan et al., 2013). Thus, TSR processes may represent important confounders to consider when assessing intervention effectiveness. For example, if self‐monitoring/working‐memory capacity influences participant responses to a self‐monitoring intervention, then understanding this relationship is vital to understanding the true treatment effect. For this reason, the current review echoes sentiments to focus on mechanisms of action within experimental studies (Hagger et al., 2020), and especially the inclusion of TSR constructs in intervention studies that target TSR‐related processes.

### 4.5 Conclusion

This scoping review aimed to explore the relationship between top‐down self‐regulatory processes and oral hygiene self‐care behavior. It found that interdental cleaning appears to depend on TSR more than toothbrushing and that there appears to be value in the use of self‐monitoring‐focused interventions to improve interdental cleaning frequency. The review recommends that the task‐difficulty of interdental cleaning be investigated to explain greater reliance on executive resources, and that future studies aim to employ more objective measures of quantifying TSR processes, especially within intervention studies that target these constructs. TSR processes represent a promising research path and their continued application is expected to improve current explanations of oral hygiene habits and contribute to the continual improvement of behavior change strategies within the oral health sector.

## CONFLICT OF INTERESTS

The authors declare no conflict of interest.

## AUTHOR CONTRIBUTIONS

Adam A. Rogers, Tiril Willumsen, Hilde Strømme, and Jan‐Are K. Johnsen developed the idea, the research question, and the search protocol and inclusion criteria. Adam A. Rogers and Hilde Strømme conducted the abstract screening. Adam A. Rogers, Tiril Willumsen, and Jan‐Are K. Johnsen all read the full‐text articles and discussed and contributed to the eventual analysis and the main direction and findings within the manuscript. All authors contributed and provided input regarding the final version of the manuscript.

## Supporting information

Supporting information.

## Data Availability

The sharing of data is not applicable to this study as no new data were created.

## APPENDIX A DIGITAL SEARCH STRATEGY

Top‐down self‐regulation processes as determinants of oral hygiene self‐care behavior: A systematic scoping review.

Search strategy

CINAHL (EBSCO) (Truncation: *, Wildcard: #, Proximity: W*n*)

Date of search: 02.04.2020

Total results: 528

**Table:**

1	MH “self‐regulation”	6721
2	(self W0 (regulat* OR monitor OR control* OR analy* OR conscious OR correcti* OR criti* OR disciplin* OR evaluat* OR judg* OR manag* OR observ* OR reflect* OR restr*))	34,651
3	(“action control” OR agency OR attention OR autoregulation OR “behavio#ral disinhibition” OR “behavio#ral inhibition” OR “behavio#ral regulat*” OR “cognitive control” OR “cognitive shifting” OR (delay* N2 gratification*) OR “executive control” OR “executive funct*” OR “focus#ed atten*” OR “impulse control” OR “inhibitory control” OR introspect* OR metacogniti* OR mindful* OR reflective* OR “response inhibition” OR "set‐shifting" OR “social comparison” OR “task switching” OR volition* OR willpower OR “working memory")	246,738
4	S1 OR S2 OR S3	276,740
5	MH “oral health"	12,718
6	(“oral health” OR “oral hygiene” OR “plaque control” OR brushing OR dental OR teeth OR tooth* OR floss*)	149,200
7	S5 OR S6	149,200
8	MH “health behavior"	109,506
9	(behavio#r* OR frequenc* OR "self‐care")	628,231
10	S8 OR S9	673,145
11	S4 AND S7 AND S10	528

Cochrane Library (Truncation: *, Wildcard: *, Proximity: NEXT)

Date of search: 02.04.2020

Total results: 179

**Table:**

1	MeSH descriptor: [Self‐Control] explode all trees	222
2	(self NEXT (regulat* OR monitor OR control* OR analy* OR conscious OR correcti* OR criti* OR disciplin* OR evaluat* OR judg* OR manag* OR observ* OR reflect* OR restr*)):ti,ab,kw	13,627
3	(“action control” OR agency OR attention OR autoregulation OR (behavio*ral NEXT *inhibition) OR (behavio*ral NEXT regulat*) OR “cognitive control” OR “cognitive shifting” OR (delay* NEAR/2 gratification*) OR “executive control” OR (executive NEXT funct*) OR (focus*ed NEXT atten*) OR “impulse control” OR “inhibitory control” OR introspect* OR metacogniti* OR mindful* OR reflective* OR “response inhibition” OR (set NEXT shifting)OR “social comparison” OR “task switching” OR volition* OR willpower OR “working memory"):ti,ab,kw	45,106
4	#1 OR #2 OR #3	56,888
5	MeSH descriptor: [Oral Health] explode all trees	383
6	(“oral health” OR “oral hygiene” OR “plaque control” OR brushing OR dental OR teeth OR tooth* OR floss*):ti,ab,kw	36,011
7	#5 OR #6	36,011
8	MeSH descriptor: [Health Behavior] explode all trees	34,433
9	(behavio*r* OR frequenc* OR (self NEXT care)):ti,ab,kw	184,765
10	#8 OR #9	207,125
11	#4 AND #7 AND #10	179

EMBASE (Ovid) (Truncation: *, Wildcard: #, Proximity: adj *n*)

Date of search: 02.04.2020

Total results: 1135

**Table:**

1	self control/	7334
2	autoregulation/	15,909
3	(self adj1 (regulat* or monitor or control* or analy* or conscious or correcti* or criti* or disciplin* or evaluat* or judg* or manag* or observ* or reflect* or restr*)).tw,kw.	72,801
4	(action control or agency or attention or autoregulation or behavio?ral disinhibition or behavio?ral inhibition or behavio?ral regulat* or cognitive control or cognitive shifting or (delay* adj3 gratification*) or executive control or executive funct* or focus?ed atten* or impulse control or inhibitory control or introspect* or metacogniti* or mindful* or reflective* or response inhibition or set‐shifting or social comparison or task switching or volition* or willpower or working memory).tw,kw.	738,261
5	1 or 2 or 3 or 4	809,195
6	dental health/	3923
7	(oral health or oral hygiene or plaque control or brushing or dental or teeth or tooth* or floss*).tw,kw.	384,157
8	6 or 7	384,747
9	health behavior/	65,192
10	(behavio?r* OR frequenc* OR self‐care).tw,kw.	2,647,385
11	9 or 10	2,671,025
12	5 and 8 and 11	1135

MEDLINE (Ovid) (Truncation: *, Wildcard: ?, Proximity: adj *n*)

Date of search: 02.04.2020

Total results: 989

**Table:**

1	self‐control/or emotional regulation/	2380
2	(self adj1 (regulat* or monitor or control* or analy* or conscious or correcti* or criti* or disciplin* or evaluat* or judg* or manag* or observ* or reflect* or restr*)).tw,kw,kf.	54,418
3	(action control or agency or attention or autoregulation or behavio?ral disinhibition or behavio?ral inhibition or behavio?ral regulat* or cognitive control or cognitive shifting or (delay* adj3 gratification*) or executive control or executive funct* or focus?ed atten* or impulse control or inhibitory control or introspect* or metacogniti* or mindful* or reflective* or response inhibition or set‐shifting or social comparison or task switching or volition* or willpower or working memory).tw,kw,kf.	544,906
4	1 or 2 or 3	593,322
5	oral health/	16,213
6	(oral health or oral hygiene or plaque control or brushing or dental or teeth or tooth* or floss*).tw,kw,kf.	361,018
7	5 or 6	363,107
8	health behavior/	49,075
9	(behavio?r* OR frequenc* OR self‐care).tw,kw,kf.	2,111,381
10	8 or 9	2,130,734
11	4 and 7 and 10	989

PsycINFO (Ovid) (Truncation: *, Wildcard: ?, Proximity: adj *n*)

Date of search: 02.04.2020

Total results: 232

**Table:**

1	self‐regulation/or agency/or emotional regulation/or self‐control/or self‐management/or self‐monitoring/or "self‐monitoring (personality)"/	41,274
2	(self adj1 (regulat* or monitor or control* or analy* or conscious or correcti* or criti* or disciplin* or evaluat* or judg* or manag* or observ* or reflect* or restr*)).ti,ab,id.	67,077
3	(action control or agency or attention or autoregulation or behavio?ral disinhibition or behavio?ral inhibition or behavio?ral regulat* or cognitive control or cognitive shifting or (delay* adj3 gratification*) or executive control or executive funct* or focus?ed atten* or impulse control or inhibitory control or introspect* or metacogniti* or mindful* or reflective* or response inhibition or set‐shifting or social comparison or task switching or volition* or willpower or working memory).ti,ab,id.	405,450
4	1 or 2 or 3	472,179
5	oral health/or exp dental health/or exp “teeth (anatomy)"/	1961
6	(oral health or oral hygiene or plaque control or brushing or dental or teeth or tooth* or floss*).ti,ab,id.	9113
7	5 or 6	9291
8	health behavior/	27,627
9	(behavio?r* OR frequenc* OR self‐care).ti,ab,id.	1,119,652
10	8 or 9	1,126,927
11	4 and 7 and 10	232

Scopus (Truncation: *, Wildcard: *, Proximity: PRE/*n*)

Date of search: 02.04.2020

Total results: 2316

**Table:**

1	TITLE‐ABS‐KEY (self PRE/0 (regulat* OR monitor or control* OR analy* or conscious OR correcti* OR criti* OR disciplin* OR evaluat* OR judg* OR manag* OR observ* OR reflect* OR restr*))	142,499
2	TITLE‐ABS‐KEY (“action control” OR agency OR attention OR autoregulation OR “behavio*ral disinhibition” OR “behavio*ral inhibition” OR “behavio*ral regulat*” OR “cognitive control” OR “cognitive shifting” OR (delay* W/2 gratificat*) OR “executive control” OR “executive funct*” OR “focus*ed atten*” OR “impulse control” OR “inhibitory control” OR introspect* OR metacogniti* OR mindful* OR reflective* OR “response inhibition” OR "set‐shifting" OR “social comparison” OR “task switching” OR volition* OR willpower OR “working memory")	1,882,904
3	#1 OR #2	2,003,293
4	TITLE‐ABS‐KEY (“oral health” OR “oral hygiene” OR “plaque control” OR brushing OR dental OR teeth OR tooth* OR floss*)	724,472
5	TITLE‐ABS‐KEY (behavio*r* OR frequenc* OR "self‐care")	7,956,200
6	#3 AND #4 AND #5	2316

Web of Science (Truncation: *, Wildcard: $, Proximity: NEAR/*n*)

Date of search: 02.04.2020

Total results: 1247

**Table:**

1	TS = (self NEAR/0 (regulat* OR monitor or control* OR analy* or conscious OR correcti* OR criti* OR disciplin* OR evaluat* OR judg* OR manag* OR observ* OR reflect* OR restr*))	110,925
2	TS = (“action control” OR agency OR attention OR autoregulation OR “behavio$ral disinhibition” OR “behavio$ral inhibition” OR “behavio$ral regulat*” OR “cognitive control” OR “cognitive shifting” OR (delay* NEAR/2 gratification*) OR “executive control” OR “executive funct*” OR “focus$ed atten*” OR “impulse control” OR “inhibitory control” OR introspect* OR metacogniti* OR mindful* OR reflective* OR “response inhibition” OR "set‐shifting" OR “social comparison” OR “task switching” OR volition* OR willpower OR “working memory")	1,255,251
3	#1 OR #2	1,347,813
4	TS = (“oral health” OR “oral hygiene” OR “plaque control” OR brushing OR dental OR teeth OR tooth* OR floss*)	362,710
5	TS = (behavio$r* OR frequenc* OR "self‐care")	5805,388
6	#3 AND #4 AND #5	1247

Total results

**Table:**

CINAHL	528
Cochrane Library	179
EMBASE	1135
MEDLINE	989
PsycINFO	232
Scopus	2316
Web of Science	1247
**Total**	**6626**

Duplicates removed via Bramer et al. method	3369
Original articles remaining and entered into Rayyan database	3257
Articles removed following title/abstract screening by AR and HS	3184
Articles to undergo full‐text screening by AR, TW, JA, AH, PK	73
Articles included in qualitative synthesis	25

## APPENDIX B DETAILS OF STUDIES EXCLUDING DURING FULL‐TEXT SCREENING

**Table:**

Author (Year)	Title of publication	Reason for exclusion
Schwarzer et al. (2007)	Adoption and maintenance of four health behaviors: Theory‐guided longitudinal studies on dental flossing, seat belt use, dietary behavior, and physical activity	Did not quantify a top‐down self‐regulatory construct. Construct defintions did to not relate enough to the executive functions defined by Miyake et al. (2000).
Dumitrescu et al. (2007)	Investigating the relationship between self‐reported oral health status, oral health‐related behaviors, type A behavior pattern, perceived stress and emotional intelligence	Did not quantify a top‐down self‐regulatory construct. Emotional Intelligence did to not relate enough to the executive functions defined by Miyake et al. (2000).
Dumitrescu et al. (2008)	Is it an association between body appreciation, self‐criticism, oral health status, and oral health‐related behaviors?	Did not quantify a top‐down self‐regulatory construct. Self‐Criticism did to not relate enough to the executive functions defined by Miyake et al. (2000).
Tedesco et al. (1992)	Effect of a Social Cognitive Intervention on Oral Health‐Status, Behavior Reports, and Cognitions	Did not quantify a top‐down self‐regulatory construct. Self‐efficacy did to not relate enough to the executive functions defined by Miyake et al. (2000).
Hamilton et al. (2017)	Translating Dental Flossing Intentions into Behavior: a Longitudinal Investigation of the Mediating Effect of Planning and Self‐Efficacy on Young Adults	Did not quantify a top‐down self‐regulatory construct. Self‐Efficacy and Planning did to not relate enough to the executive functions defined by Miyake et al. (2000).
Halvari et al. (2010)	Motivation and anxiety for dental treatment: Testing a self‐determination theory model of oral self‐care behaviour and dental clinic attendance.	Did not quantify a top‐down self‐regulatory construct. Self‐Regulation Questionnaire for Dental Treatment (SRQDT) did to not relate enough to the executive functions defined by Miyake et al. (2000).
Halvari et al. (2012)	Motivation for dental home care: Testing a self‐determination theory model.	Did not quantify a top‐down self‐regulatory construct. Self‐Regulation Questionnaire for Dental Treatment (SRQDT) did to not relate enough to the executive functions defined by Miyake et al. (2000).
Almomani (2007)	The effects of an oral health promotion program in people with serious mental illness	Did not quantify oral hygiene self‐care frequency
Almomani et al. (2009)	Effects of an oral health promotion program in people with mental illness	Did not quantify oral hygiene self‐care frequency
Hamilton et al. (2018)	Parental supervision for their children's toothbrushing: Mediating effects of planning, self‐efficacy, and action control	Did not quantify oral hygiene self‐care frequency
Hui et al. (2009)	Performance, cardiovascular, and health behavior effects of an inhibitory strength training intervention	Did not quantify oral hygiene self‐care frequency
Kimura et al. (2013)	Evaluation of chewing ability and its relationship with activities of daily living, depression, cognitive status, and food intake in the community‐dwelling elderly	Did not quantify oral hygiene self‐care frequency
Morchadze et al. (2018)	Correlation between the Oral Hygienic Condition and Psycho‐Social Factors in the Elderly Population of Imereti	Did not quantify oral hygiene self‐care frequency
Saengtipbovorn and Taneepanichskul (2015)	Lifestyle Change Plus Dental Care (LCDC) program improves knowledge, attitude, and practice (KAP) toward oral health and diabetes mellitus among the elderly with type 2 diabetes	Did not quantify oral hygiene self‐care frequency
Schaber et al. (2013)	Using Cognitive‐Functional Assessment to Predict Self‐Care Performance of Memory Care Tenants	Did not quantify oral hygiene self‐care frequency
Sharma et al. (2019)	Testing of a dental student‐administered multidisciplinary health promotion program	Did not quantify oral hygiene self‐care frequency
Simpriano and Mialhe (2017)	Impact of Educational Interventions Based on the Implementation Intentions Strategy on the Oral Health of Schoolchildren	Did not quantify oral hygiene self‐care frequency
Uskul et al. (2009)	The cultural congruency effect: Culture, regulatory focus, and the effectiveness of gain‐ versus loss‐framed health messages	Did not quantify oral hygiene self‐care frequency
Junko et al. (2012)	Development of a Self‐Control Scale Associated with Health Behavior for Older Adults in Community.	Full‐text not available in English, Norwegian, Danish or Swedish
Kawamoto (1985)	The effects of self‐evaluation and behavior standard settings on toothbrushing behavior in a preschool classroom	Full‐text not available in English, Norwegian, Danish or Swedish
Kunitsuka et al. (2002)	A correspondence behavioral approach for 6 lifestyle's improvements in a workplace	Full‐text not available in English, Norwegian, Danish or Swedish
Gholami et al. (2015)	A Brief Self‐Regulatory Intervention Increases Dental Flossing in Adolescent Girls	Intervention did not target top‐down self‐regulatory constructs. Format was deemed unlikely to train or influence the executive functions defined by Miyake et al. (2000).
Lhakhang et al. (2016)	Combining self‐management cues with incentives to promote interdental cleaning among Indian periodontal disease outpatients	Intervention did not target top‐down self‐regulatory constructs. Format was deemed unlikely to train or influence the executive functions defined by Miyake et al. (2000).
Fjellström et al. (2010)	A modified cognitive‐behavioral model as a method to improve adherence to oral hygiene instructions‐‐a pilot study.	Quantitative statistical comparison not avaiable
Gaeta et al. (2018)	Fostering Oral Hygiene Habits and Self‐Regulation Skills: An Intervention With Preschool Children	Quantitative statistical comparison not available
Jönsson et al. (2009)	An individually tailored treatment program for improved oral hygiene: Introduction of a new course of action in health education for patients with periodontitis	Quantitative statistical comparison not available
McCaul et al. (1988)	Predicting the performance of dental hygiene behaviors: An examination of the Fishbein and Ajzen model and self‐efficacy expectations.	Quantitative statistical comparison not available
Moriya et al. (2012)	Relationship between periodontal status and intellectual function among community‐dwelling elderly persons.	Quantitative statistical comparison not available
Nishihira et al. (2012)	Community oral health promotion program fostering self‐management for the elderly	Quantitative statistical comparison not available
O'Hara et al. (2008)	Using personal digital assistants to improve self‐care in oral health	Quantitative statistical comparison not available
Ojo et al. (2015)	OH‐BUDDY: Mobile phone texting based intervention for diabetes and oral health management	Quantitative statistical comparison not available
Park and Chang (2014)	Effect of a health coaching self‐management program for older adults with multimorbidity in nursing homes.	Quantitative statistical comparison not avaiable
Philippot et al. (2005)	Improving patients' compliance with the treatment of periodontitis: a controlled study of behavioral intervention.	Quantitative statistical comparison not available
Sakashita et al. (2017)	Oral health promotion program for fostering self‐management of the elderly living in communities	Quantitative statistical comparison not available
Akbarfahimi (2019)	The effect of the order of Transcranial direct current stimulation and Computer‐based Cognitive Rehabilitation on Improving Cognitive Performance and Activities of Daily Living	Study protocol
Chaly (2018)	Field Trial to improve oral health behavior through a smartphone dental app	Study protocol
Eberhard (2018)	Think Dental, Be Active!: a randomized control trial using psycho‐education, physical activity, and oral health interventions in older adults aged >50 years old, residing in Royal Freemason Benevolent Institute residential aged care facilities	Study protocol
Pakpour et al. (2016)	The Effects of Two Volitional Interventions to Improve Oral Health Behaviour Among Iranian Adolescents	Study protocol
Scheerman et al. (2018)	A Mobile App (WhiteTeeth) to Promote Good Oral Health Behavior Among Dutch Adolescents with Fixed Orthodontic Appliances: Intervention Mapping Approach	Study protocol
Bonetti et al. (2006)	Behavioral educational intervention can improve patients’ compliance with prophylaxis: Can behavioral educational interventions based on the self‐regulation theory improve periodontitis patients’ compliance with proper dental care?	Synthesis of existing data
Schwarzer (2008)	Modeling health behavior change: how to predict and modify the adoption and maintenance of health behaviors.	Synthesis of existing data
Schwarzer (2016)	Health action process approach (HAPA) as a theoretical framework to understand behavior change.	Synthesis of existing data
Schüz et al. (2005)	Stage‐specific effects of action control on regular preventive dental health behavior.	Synthesis of existing data
Yeung (2010)	Motivational interviewing in an oral health promotion program	Synthesis of existing data
Aminabadi et al. (2014)	Can child temperament be related to early childhood caries?	Used 3rd party reports for behavior/psychological measures
Dursun et al. (2016)	Mind Conduct disorders in children with poor oral hygiene habits and attention deficit hyperactivity disorder in children with excessive tooth decay.	Used 3rd party reports for behavior/psychological measures
Gilinsky et al. (2012)	Development and testing of a theory‐based behavioral change intervention: A pilot investigation in a nursery school in a deprived area of Scotland	Used 3rd party reports for behavior/psychological measures
Matsuyama et al. (2018)	Self‐control and dental caries among elementary school children in Japan	Used 3rd party reports for behavior/psychological measures

*Note*: References cited in first column are available as supporting information Appendix.
