# Supplementary material for: Top‐down self‐regulation processes as determinants of oral hygiene self‐care behaviour: A systematic scoping review
Source: Clin Exp Dent Res. 2022 Apr 9;8(4):807–26. doi: 10.1002/cre2.548 (PMC9382055; doi:10.1002/cre2.548)
Supplement: Supplementary file 1 — Supporting information. [file CRE2-8-807-s001.docx]

**References**

Akbarfahimi, M. (2019). The effect of the order of Transcranial direct current stimulation and Computer-based Cognitive Rehabilitation on Improving Cognitive Performance and Activities of Daily Living. [*http://www.who.int/trialsearch/Trial2.aspx?TrialID=IRCT20120910010806N8*](http://www.who.int/trialsearch/Trial2.aspx?TrialID=IRCT20120910010806N8).

Almomani, F. (2007). *The effects of an oral health promotion program in people withserious mental illness.* University of Kansas,

Almomani, F., Williams, K., Catley, D., & Brown, C. (2009). Effects of an oral health promotion program in people with mental illness. *Journal of dental research, 88*(7), 648-652.

Aminabadi, N., Ghoreishizadeh, A., Ghoreishizadeh, M., Oskouei, S. G., & Ghojazadeh, M. (2014). Can child temperament be related to early childhood caries? *Caries Research, 48*(1), 3-12.

Bonetti, D. (2006). Behavioural educational intervention can improve patients’ compliance with prophylaxis: Can behavioural educational interventions based on the self-regulation theory improve periodontitis patients’ compliance with proper dental care? *Evidence-based dentistry, 7*(1), 11.

Chaly, P. E. (2018). Field Trial to improve oral health behavior through a smart phone dental app. [*http://www.who.int/trialsearch/Trial2.aspx?TrialID=CTRI/2018/12/016786*](http://www.who.int/trialsearch/Trial2.aspx?TrialID=CTRI/2018/12/016786).

Dumitrescu, A. L. (2007). Investigating the relationship between self-reported oral health status, oral health-related behaviors, type A behavior pattern, perceived stress and emotional intelligence. *Romanian journal of internal medicine = Revue roumaine de medecine interne, 45*(1), 67-76.

Dumitrescu, A. L., Zetu, L., Teslaru, S., Dogaru, B. C., & Dogaru, C. D. (2008). Is it an association between body appreciation, self-criticism, oral health status and oral health-related behaviors? *Romanian journal of internal medicine = Revue roumaine de medecine interne, 46*(4), 343-350.

Dursun, O. B., Esin, Ý. S., Yücel, N., Şengül, F., Demirci, T., Ömezli, M. M., & Esin, İ. S. (2016). Mind Conduct disorders in children with poor oral hygiene habits and attention deficit hyperactivity disorder in children with excessive tooth decay. *Archives of Medical Science, 12*(6), 1279-1285.

Eberhard, J. (2018). Think Dental, Be Active!: a randomised control trial using psycho-education, physical activity and oral health interventions in older adults aged >50 years old, residing in Royal Freemason Benevolent Institute residential aged care facilities. [*http://www.who.int/trialsearch/Trial2.aspx?TrialID=ACTRN12618001599268*](http://www.who.int/trialsearch/Trial2.aspx?TrialID=ACTRN12618001599268).

Fjellström, M., Yakob, M., & Söder, B. (2010). A modified cognitive behavioural model as a method to improve adherence to oral hygiene instructions–a pilot study. *International journal of dental hygiene, 8*(3), 178-182.

Gaeta, M. L., Cavazos, J., Cabrera, M. D. R., & Rosario, P. (2018). Fostering Oral Hygiene Habits and Self-Regulation Skills: An Intervention With Preschool Children. *Family & community health, 41*(1), 47-54. doi:10.1097/FCH.0000000000000171

Gholami, M., Knoll, N., & Schwarzer, R. (2015). A Brief Self-Regulatory Intervention Increases Dental Flossing in Adolescent Girls. *International journal of behavioral medicine, 22*(5), 645-651. doi:10.1007/s12529-014-9459-6

Gilinsky, A., Swanson, V., Merrett, M., Power, K., & Marley, L. (2011). Development and testing of a theory-based behavioural change intervention: A pilot investigation in a nursery school in a deprived area of Scotland. *Community dental health, 29*(1), 62-67.

Halvari, A. E. M., Halvari, H., Bjornebekk, G., & Deci, E. L. (2010). Motivation and anxiety for dental treatment: Testing a self-determination theory model of oral self-care behaviour and dental clinic attendance. *Motivation and Emotion, 34*(1), 15-33.

Halvari, A. E. M., Halvari, H., Bjornebekk, G., & Deci, E. L. (2012). Motivation for dental home care: Testing a self-determination theory model. *Journal of Applied Social Psychology, 42*(1), 1-39.

Hamilton, K., Bonham, M., Bishara, J., Kroon, J., & Schwarzer, R. (2017). Translating Dental Flossing Intentions into Behavior: a Longitudinal Investigation of the Mediating Effect of Planning and Self-Efficacy on Young Adults. *International journal of behavioral medicine, 24*(3), 420-427.

Hamilton, K., Cornish, S., Kirkpatrick, A., Kroon, J., & Schwarzer, R. (2018). Parental supervision for their children's toothbrushing: Mediating effects of planning, self‐efficacy, and action control. *British Journal of Health Psychology, 23*(2), 387-406.

Hui, S. k. A., Wright, R. A., Stewart, C. C., Simmons, A., Eaton, B., & Nolte, R. N. (2009). Performance, cardiovascular, and health behavior effects of an inhibitory strength training intervention. *Motivation and Emotion, 33*(4), 419-434.

Jonsson, B., Ohrn, K., Oscarson, N., & Lindberg, P. (2009). An individually tailored treatment programme for improved oral hygiene: introduction of a new course of action in health education for patients with periodontitis. *International journal of dental hygiene, 7*(3), 166-175. doi:10.1111/j.1601-5037.2008.00350.x

Junko, F., Yayoi, K., Takayuki, S., Yumiko, M., Maya, N., Ayumi, F., & Yukari, Y. (2012). Development of a Self-Control Scale Associated with Health Behavior for Older Adults in Community. *Journal of Japan Academy of Nursing Science, 32*(3), 85-93.

Kawamoto, H. (1985). The effects of self-evaluation and behavior standard settings on toothbrushing behavior in a preschool classroom. *Japanese Journal of Educational Psychology, 33*(4), 307-314.

Kimura, Y., Ogawa, H., Yoshihara, A., Yamaga, T., Takiguchi, T., Wada, T., . . . Matsubayashi, K. (2013). Evaluation of chewing ability and its relationship with activities of daily living, depression, cognitive status and food intake in the community-dwelling elderly. *Geriatrics & Gerontology International, 13*(3), 718-725.

Kunitsuka, K., Yamatsu, K., & Adachi, Y. (2002). [A correspondence behavioral approach for 6 lifestyle's improvements in a workplace]. *[Nihon koshu eisei zasshi] Japanese journal of public health, 49*(6), 525-534.

Lhakhang, P., Hamilton, K., Sud, N., Sud, S., Kroon, J., Knoll, N., & Schwarzer, R. (2016). Combining self-management cues with incentives to promote interdental cleaning among Indian periodontal disease outpatients. *BMC oral health, 16*(1), 6.

Matsuyama, Y., Fujiwara, T., Ochi, M., Isumi, A., & Kato, T. (2018). Self‐control and dental caries among elementary school children in Japan. *Community Dentistry & Oral Epidemiology, 46*(5), 465-471.

McCaul, K. D., O'Neill, H. K., & Glasgow, R. E. (1988). Predicting the Performance of Dental Hygiene Behaviors: An Examination of the Fishbein and Ajzen Model and Self‐Efficacy Expectations 1. *Journal of Applied Social Psychology, 18*(2), 114-128.

Morchadze, L., Margvelashvili, V., Taboridze, I., & Aladashvili, L. (2018). Correlation between the Oral Hygienic Condition and Psycho-Social Factors in the Elderly Population of Imereti. *Georgian medical news*(274), 48-51.

Moriya, S., Tei, K., Toyoshita, Y., Koshino, H., Inoue, N., & Miura, H. (2012). Relationship between periodontal status and intellectual function among community-dwelling elderly persons. *Gerodontology, 29*(2), e368-e374.

Nishihira, T., Nishitani, M., Sato, T., Abiko, Y., Matsushita, K., Hamada, M., . . . Sakashita, R. (2012). Community oral health promotion program fostering self-management for the elderly. *Interface Oral Health Science 2011*, 317-318.

O'Hara, D. M., Seagriff-Curtin, P., Levitz, M., Davies, D., & Stock, S. (2008). Using personal digital assistants to improve self-care in oral health. *Journal of Telemedicine & Telecare, 14*(3), 150-151.

Ojo, A., Chatterjee, S., Neighbors, H. W., Piatt, G. A., Moulik, S., Neighbors, B. D., . . . Jones, D. (2015). OH-BUDDY: Mobile phone texting based intervention for diabetes and oral health management. *Proceedings of the Annual Hawaii International Conference on System Sciences, 2015*, 803-813.

Pakpour, A. (2016). The Effects of Two Volitional Interventions to Improve Oral Health Behaviour Among Iranian Adolescents. [*https://clinicaltrials.gov/show/NCT02066987*](https://clinicaltrials.gov/show/NCT02066987).

Park, Y. H., & Chang, H. (2014). Effect of a health coaching self-management program for older adults with multimorbidity in nursing homes. *Patient preference and adherence, 8*, 959-970.

Philippot, P., Lenoir, N., D'Hoore, W., & Bercy, P. (2005). Improving patients' compliance with the treatment of periodontitis: a controlled study of behavioural intervention. *Journal of clinical periodontology, 32*(6), 653-658.

Saengtipbovorn, S., & Taneepanichskul, S. (2015). Lifestyle Change Plus Dental Care (LCDC) program improves knowledge, attitude, and practice (KAP) toward oral health and diabetes mellitus among the elderly with type 2 diabetes. *Journal of the Medical Association of Thailand = Chotmaihet thangphaet, 98*(3), 279-290.

Sakashita, R., Hamada, M., Sato, T., Abiko, Y., & Takami, M. (2017). Oral health promotion program for fostering self-management of the elderly living in communities. *Intelligent Automation and Soft Computing, 23*(3), 535-541.

Schaber, P., Klein, T., Hanrahan, E., Vencil, P., Afatika, K., & Burns, T. (2013). Using cognitive-functional assessment to predict self-care performance of memory care tenants. *American Journal of Alzheimer's Disease & Other Dementias®, 28*(2), 171-178.

Scheerman, J. F. M. (2017). The WhiteTeeth app to promote oral hygiene. [*http://www.who.int/trialsearch/Trial2.aspx?TrialID=NTR6206*](http://www.who.int/trialsearch/Trial2.aspx?TrialID=NTR6206).

Schwarzer, R. (2008). Modeling health behavior change: How to predict and modify the adoption and maintenance of health behaviors. *Applied psychology, 57*(1), 1-29.

Schwarzer, R. (2016). Health action process approach (HAPA) as a theoretical framework to understand behavior change. *Actualidades en Psicologia, 30*(121), 119-130.

Schwarzer, R., Schüz, B., Ziegelmann, J. P., Lippke, S., Luszczynska, A., & Scholz, U. (2007). Adoption and maintenance of four health behaviors: Theory-guided longitudinal studies on dental flossing, seat belt use, dietary behavior, and physical activity. *Annals of Behavioral Medicine, 33*(2), 156-166.

Schüz, B., Sniehotta, F., Wiedemann, A., & Seemann, R. (2005). Stage-specific effects of action control on regular preventive dental health behaviour. *Psychology & Health, 20*, 240-240.

Sharma, A., Jain, M., Singh, S., Yadav, N. R., Chahar, P., Monga, A., & Jain, V. (2019). Testing of a dental student-administered multidisciplinary health promotion program. *Journal of family medicine and primary care, 8*(10), 3230-3235.

Simpriano, A. B., & Luiz, F. (2017). Impact of educational interventions based on the implementation intentions strategy on the oral health of schoolchildren. *Pesquisa Brasileira em Odontopediatria e Clínica Integrada*.

Tedesco, L. A., Keffer, M. A., Davis, E. L., & Christersson, L. A. (1992). Effect of a Social Cognitive Intervention on Oral Health-Status, Behavior Reports, and Cognitions. *Journal of Periodontology, 63*(7), 567-575.

Uskul, A. K., Sherman, D. K., & Fitzgibbon, J. (2009). The cultural congruency effect: Culture, regulatory focus, and the effectiveness of gain- vs. loss-framed health messages. *Journal of Experimental Social Psychology, 45*(3), 535-541.

Yeung, C. A. (2010). Motivational interviewing in an oral health promotion programme. *Evidence-based dentistry, 11*(1), 14-15. doi:10.1038/sj.ebd.6400703
